# *Drosophila tafazzin* mutants have impaired exercise capacity

**DOI:** 10.14814/phy2.13604

**Published:** 2018-02-06

**Authors:** Deena Damschroder, Christian Reynolds, Robert Wessells

**Affiliations:** ^1^ Deparment of Physiology Wayne State University School of Medicine Detroit Michigan; ^2^ Department of Emergency Medicine Wayne State University School of Medicine Detroit Michigan

**Keywords:** Barth syndrome, *drosophila*, exercise intolerance, *tafazzin*

## Abstract

Cardiolipin (CL) is a mitochondrial phospholipid that helps maintain normal structure of the inner mitochondrial membrane and stabilize the protein complexes of the electron transport chain to promote efficient ATP synthesis. Tafazzin, an acyl‐transferase, is required for synthesis of the mature form of CL. Mutations in the *tafazzin (TAZ)* gene are associated with a human disorder known as Barth syndrome. Symptoms of Barth syndrome often include muscle weakness and exercise intolerance. Previous work demonstrates that *Drosophila Taz* mutants exhibit motor weakness, as measured by reduced flying and climbing abilities. However, *Drosophila TAZ* mutants’ baseline endurance or response to endurance exercise training has not been assessed. Here, we find that *TAZ* mutants have reduced endurance and do not improve following a stereotypical exercise training paradigm, indicating that loss of *TAZ* function leads to exercise intolerance in *Drosophila*. Although cardiac phenotypes are observed in human Barth syndrome patients, *TAZ* mutants had normal resistance to cardiac pacing. In the future, endurance may be a useful screening tool to identify additional genetic modifiers of *tafazzin*.

## Introduction

Cardiolipin (CL) is a dimeric phospholipid found uniquely in the mitochondria and is essential for proper mitochondrial function (Schlame et al. 2000; Schlame and Ren 2009; Xu et al. 2016). CL is enriched within the inner mitochondrial membrane, where it helps to stabilize the protein complexes of the electron transport chain, and plays a role in apoptosis through its interactions with cytochrome C. (Fry and Green 1980; Schlame and Haldar 1993; Xu et al. 2016). The synthesis of CL is a multistep process that occurs entirely within the mitochondria (Hostetler et al. 1971; Hostetler and Van den Bosch 1972). Phosphatidic acid (PA) is first converted to cytidine diphosphate diacylglycerol (CDP‐DAG) in the inner mitochondrial membrane (Hostetler et al. 1971). CDP‐DAG is then converted into phosphatidylglycerol phosphate (PGP) by PGP synthase (Chang and Kennedy 1967; Hostetler and Van den Bosch 1972). Next, PGP is dephosphorylated to create phosphatidylglycerol (PG) and an additional CDP‐DAG molecule is bound to PG by cardiolipin synthase to form one molecule of CL containing two phosphatidic acid moieties and four acyl chains (Chang et al. 1998; Chen et al. 2006).

Newly synthesized CL is extensively remodeled to obtain CL molecules with specific acyl chain combinations, and defective CL remodeling leads to Barth syndrome in humans (Schlame et al. 2000). In general, remodeled CL contains predominately unsaturated acyl chains. However, the exact acyl chain combination present in remodeled CL is species and tissue specific (Schlame and Greenberg 2017). Remodeling of CL begins with the removal of an acyl‐chain leading to the formation of monolyso‐CL (MLCL) intermediate (Schlame and Rustow 1990). In yeast, this reaction is catalyzed by the cardiolipin‐specific, phospholipase, *Cld1p,* (Beranek et al. 2009). While no cardiolipin‐specific phospholipase has been identified in flies or mammals, calcium‐independent phospholipase A2 enzymes likely contribute to cardiolipin deacylation in these species (Malhotra et al. 2009; Liu et al. 2017).

Tafazzin is a transacylase that functions to re‐acylate MLCL (Xu et al. 2006b). Tafazzin's enzymatic activity transfers an acyl group from a donor phospholipid to a lysophospholipid (Xu et al. 2006b). Tafazzin can interact with many phospholipid and lysophospholipid species and has generally been described as lacking acyl chain specificity (Schlame et al. 2012b; Schlame 2013). However, a more recent study suggests that tafazzin may discriminate based on the molecular configuration of the acyl chains of the donor phospholipid (Abe et al. 2017). Tafazzin‐dependent remodeling of CL, leading to the incorporation of unsaturated acyl residues, likely contributes to stabilizing the curvature of the inner mitochondrial membrane (Schlame et al. 2012a).

The human tafazzin gene is found on the X‐chromosome (Bione et al. 1996) and has conserved homologs ranging from single celled organisms throughout the eukaryotes (Gu et al. 2004; Xu et al. 2006a; Phoon et al. 2012). Barth syndrome is clinically characterized by neutropenia, cardiomyopathy, skeletal muscle weakness, and delayed growth (Spencer et al. 2006). Patients with Barth syndrome are often diagnosed with exercise intolerance (Spencer et al. 2011), with decreased endurance and increased time to recovery. There is currently no cure for this syndrome; only symptom management is available.

There are various models available to study Barth syndrome, including yeast and *Drosophila melanogaster*. Here, we examine the exercise capacity of *Drosophila tafazzin* mutants (*TAZ* ^−/−^). Previous work found that *TAZ* was localized to the mitochondria (Schlame 2013). Western blotting showed that *TAZ* ^−/−^ flies lack full length *tafazzin* (Xu et al. 2006a) and mutants have a reduced ability to climb and to fly (Xu et al. 2006a).

Here, we examined speed, endurance and flight of *TAZ* mutants across ages, with and without exercise training. *TAZ* mutants exhibited significantly reduced climbing speed, endurance, and flight ability compared to control (*TAZ* ^+/+^) flies. They also failed to improve their performance after a 3‐week chronic endurance exercise program. The exercise intolerance of *TAZ* ^−/−^ flies parallels that of many Barth syndrome patients, and indicates these flies could be used in future studies to further elucidate the mechanisms of exercise intolerance associated with *tafazzin* mutations. Additionally, the endurance phenotype identified in this study has the potential to be used as a high‐throughput, low‐cost assay for future genetic or pharmacological screens to find new interactors with *tafazzin*.

## Materials and Methods

### Fly lines and care

*Tafazzin* mutant flies (*TAZ* ^−/−^), w;DTAZ/CyoGFP, were created by imprecise excision of the p‐element KG02529, and lack expression of the full‐length version of *tafazzin* (Xu et al. 2006a). The control flies (*TAZ* ^+/+^), w;ΔKG02529, are the same back ground as *TAZ* ^−/−^ flies and only differ in the fact that they have a precise excision of the p‐element, causing no mutation in *tafazzin*. Age‐matched male flies were kept in vials of 20 on a twelve‐hour light/dark cycle in a 25‐degree incubator. Monday through Friday, flies were flipped onto fresh food comprised of 10% sugar and 10% yeast unless specifically noted. Male flies were used for this study because males have a much higher exercise capacity and exercise response than females (Sujkowski et al. 2015).

### Lifespan

Male progeny were collected over a 24‐h time period. Food vials were changed daily Monday through Friday. At that time, the number of dead flies was recorded. Flies were kept at 25° Celsius on a twelve‐hour light/dark cycle. Significance was tested by a log‐rank test.

### Climbing speed

Climbing speed was assessed using the Rapid Iterative Negative Geotaxis (RING) assay (Gargano et al. 2005). On day one of the experiment, a group of twenty flies were gravity transferred to a polypropylene vial, which was then placed in the RING apparatus. Five vials per genotype were tested, equaling 120 flies total. The apparatus was then swiftly tapped on the table three times to elicit negative geotaxis. A photo was taken 2 sec after the last tap to capture the height at which the flies climbed to. A total of four pictures were taken for each genotype, and the four assessments were averaged. This experiment lasted 5 weeks with flies being tested five times per week in order to assess the decline in climbing speed with age longitudinally.

ImageJ was used to analyze the pictures and determine the relative climbing height of the flies. These data were placed into Microsoft Excel and the climbing height was translated into quadrants. The data were normalized to the first 4 days and plotted as a function of the starting climbing height. Significance was assessed by two‐way ANOVA with Bonferroni correction for multiple tests.

### Endurance

Endurance was assessed using an automated negative geotaxis machine known as the Power Tower (Piazza et al. 2009), which continuously lifted and dropped vials of flies until shut off. The dropping motion triggers negative geotaxis repetitively. Eight vials of twenty flies were placed on the machine for each genotype. Vials were then visually scored for fatigue, which was determined when five or fewer flies were able to climb higher than 2 cm after three consecutive drops of the machine (Tinkerhess et al. 2012).

### Flight assay

The flight assay was performed at room temperature and as described by (Babcock and Ganetzky 2014). Briefly, flies were gravity dropped into a drop tube which released them into a cylinder that was coated with adhesive material. The drop triggered the fly's natural instinct to start flying and flies with better flight ability landed higher. Afterward, a picture was taken to capture where the flies landed and ImageJ was used to analyze the landing height of the flies. An unpaired *t*‐test was used to test for significance.

### Cardiac pacing

Flies were anesthetized with FlyNap (Carolina Biological) and placed on a modified microscope slide on a temperature‐controlled stage connected to a square‐wave stimulator. Each fly was paced for 30 sec to 6 Hz, about twice the resting heart rate at room temperature. After 30 sec, current was stopped and the response of the heart was visually scored (Wessells and Bodmer 2004). Hearts were scored as failing if they were in arrest or fibrillation, and scored as not failing if they resumed normal heartbeat. The percentage of flies that entered failure after pacing is reported as failure rate. Flies were tested at 1 week old and 5 weeks old. Significance was tested by an *F*‐test for binary variables.

### Endurance training protocol

Male *TAZ* ^−/−^ flies (*TAZ* ^−/−^ EX) were exercised as described (Piazza et al. 2009). For 3 weeks, flies were exercised daily (Monday through Friday) on the Power Tower. The time on the machine was increased by half an hour weekly, with the maximum amount of time peaking at 180 min per day. Unexercised aged‐matched control flies (*TAZ* ^−/−^ UN) were placed on the machine next to *TAZ* ^−/−^ EX flies with the only difference being a soft cap was pushed down so that the *TAZ* ^−/−^ UN could not climb and exercise. After the third week, endurance, flight ability, and cardiac stress performance was analyzed as described above.

## Results

### TAZ^−/−^ mutants have normal lifespan

**Figure 1:**
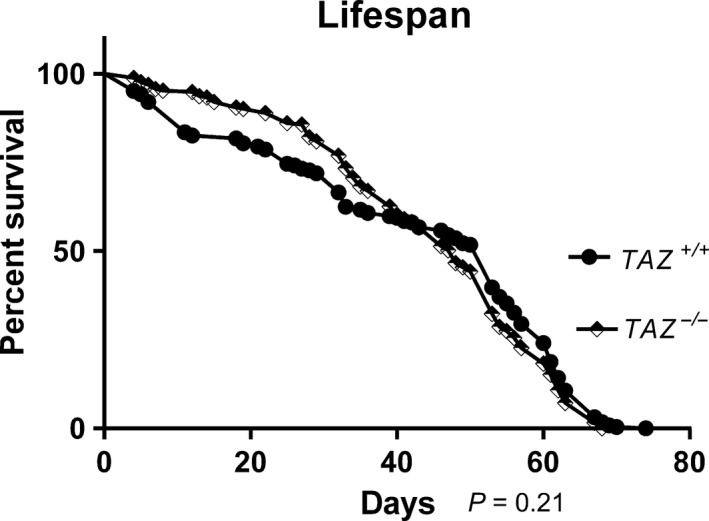
The lifespan of *TAZ* ^−/−^ and *TAZ* ^+/+^ flies were not significantly different. The median survival for *TAZ* ^+/+^ (black circles, *n* = 260) was 53 days and the median survival for *TAZ* ^−/−^ flies (black and white diamonds, *n* = 260) was 48 days. A log‐rank analysis found no significant difference between these lifespan curves (*P* = 0.21).

The lifespans of *TAZ* ^+/+^ and *TAZ* ^−/−^ males were not significantly different at 25°C on standard 10% sucrose 10% yeast diet (Fig. 1), as has been previously reported (Xu et al. 2006a).

### The climbing speed, endurance, and flight performance of TAZ^−/−^ mutants is significantly reduced

**Figure 2:**
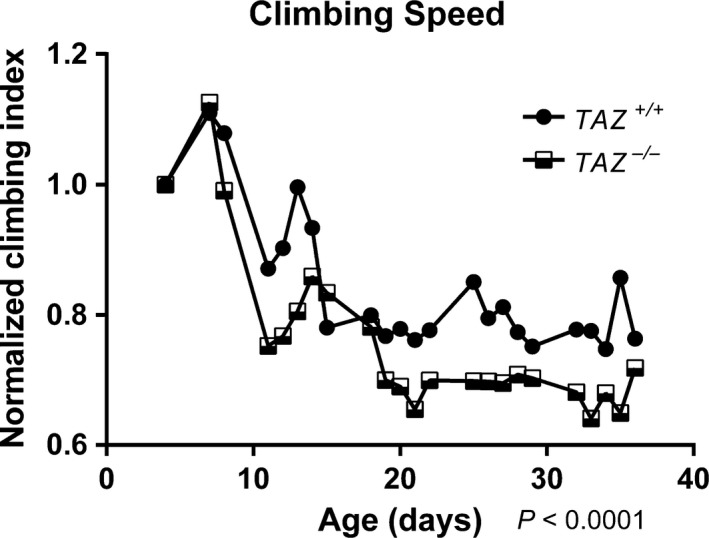
*TAZ* ^−/−^ flies have reduced climbing speed relative to *TAZ* ^+/+^ flies. Both *TAZ* ^−/−^ (black and white squares, *n* = 100) and *TAZ* ^+/+^ (black circles, *n* = 100) flies climbing speed declined with age (Two‐way ANOVA,* P* < 0.0001 Overall, the climbing speed between TAZ ^‐/‐^ and TAZ ^+/+^ flies was significantly different across the 5 weeks (2‐way ANOVA with a Bonferroni correction for multiple comparisons, *P* <  0.0001). The number at the start of this experiment was 100 for both genotypes.

Over the course of 5 weeks, an age‐related decline in climbing speed was observed in both *TAZ* ^−/−^ flies and *TAZ* ^+/+^ flies (Fig. 2). In addition, there was a significant difference in climbing speed across the 5 week measurement period between *TAZ* ^−/−^ and *TAZ* ^+/+^ flies (Fig. 2).

### TAZ^−/−^ mutants have reduced endurance and flight ability

**Figure 3:**
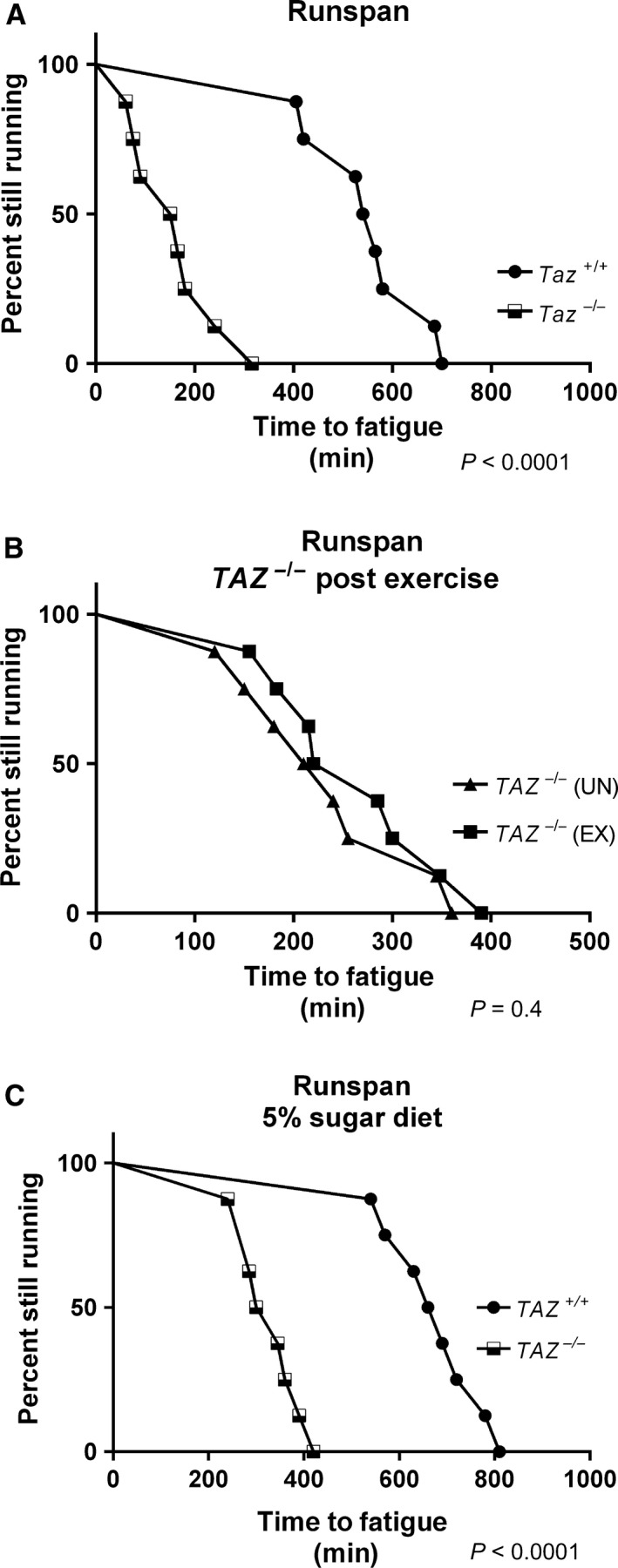
*TAZ* ^−/−^ flies have reduced endurance. (A) There is a significant difference in time to fatigue between *TAZ* ^−/−^ flies (black and white squares) and *TAZ* ^+/+^ flies (black circles) *P* < 0.0001, *n* = 160 for both groups). (B) After endurance training, there was no significant difference between *TAZ* ^−/−^ flies that were exercised (black squares) versus *TAZ* ^−/−^ flies that were unexercised (black triangles) (*P* = 0.4). (C) This significant difference remains when *TAZ* ^−/−^ (black and white squares) flies and *TAZ* ^+/+^ (black circles) flies are fed a low sugar diet (*P* < 0.0001, *n* = 160). Significance was calculated using a log‐rank test.

**Figure 4:**
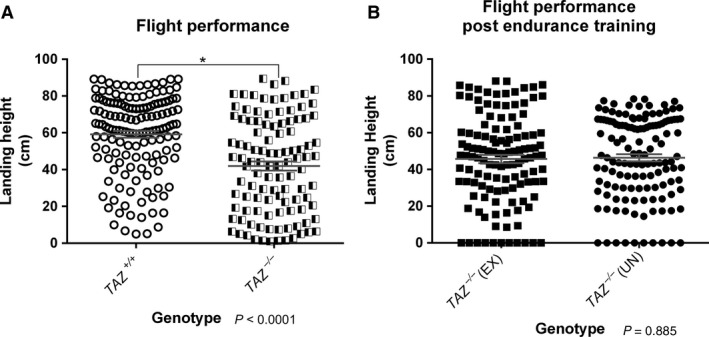
*TAZ* ^−/−^ flies have reduced flight ability. (A) The average landing height of *TAZ* ^+/+^ flies (black circles, 59.19 cm, *n* = 146) was significantly higher than the average landing height for *TAZ* ^−/−^ flies (half‐filled diamonds, 41.95 cm, *n* = 108) (*P* < 0.0001). (B) The average landing height of *TAZ* ^−/−^ EX (black squares, 46.38 cm, *n* = 113) and *TAZ* ^−/−^ UN (black circles, 45.68 cm, *n* = 123) was not significant (*P* = 0.885) as calculated by an unpaired *T*‐test.

*TAZ* ^−/−^ flies fatigued sooner than *TAZ* ^+/+^ flies (Fig. 3A), indicating a significantly reduced endurance capacity. *TAZ* ^*‐/ ‐*^ flies had significantly reduced flight performance, as measured by average landing height (Fig. 4A). This reduction in flight ability was also seen by the Schlame lab (Xu et al. 2006a).

### TAZ^−/−^ mutants’ endurance and flight ability do not improve with exercise

Wild‐type flies consistently respond to a 3‐week exercise program with improvements to endurance and flight performance (Sujkowski et al. 2015, 2017). After 3 weeks of endurance exercise training, *TAZ* mutants (*TAZ* ^−/−^ EX) did not run significantly longer than unexercised mutant controls (*TAZ* ^−/−^ UN) (Fig. 3B), demonstrating that mutants did not improve endurance with training. Similarly, the flight performance of exercised *TAZ* ^−/−^ EX was not significantly better than unexercised *TAZ* ^−/−^ UN (Fig. 4B).

### A reduced sugar diet does not exacerbate the exercise intolerance of TAZ^−/−^ mutants

Due to evidence that low‐sugar media is detrimental to CL knockout yeast (Raja et al. 2017), we also measured the runspan of *TAZ* ^+/+^ and *TAZ* ^−/−^ flies fed a 5% glucose diet. Reduced glucose availability had no detrimental effect on mutant endurance (Fig. 3C). Instead, both mutant and control flies ran somewhat longer than on 10% glucose diet, as we have previously seen in wild‐type animals (Bazzell et al. 2013). The relative difference between *TAZ* ^−/−^ and *TAZ* ^+/+^ endurance was the same on either diet (compare Fig. 3A–C).

### Taz mutants have normal response to cardiac pacing

**Figure 5:**
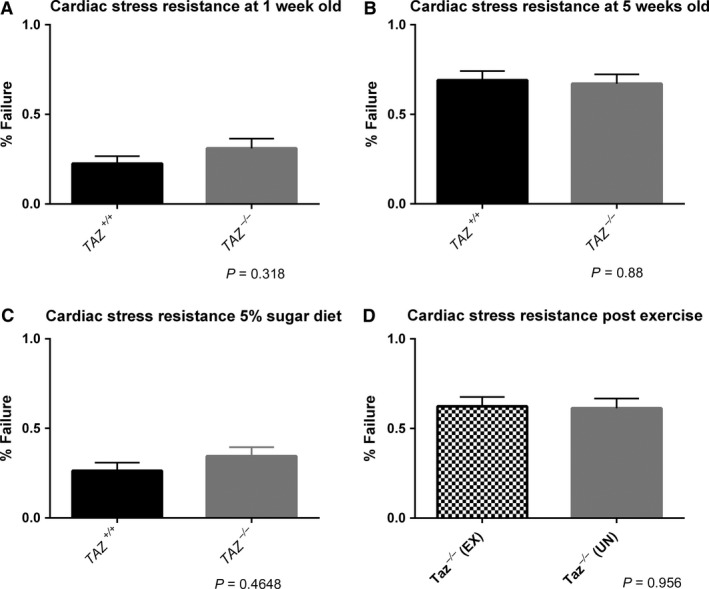
*TAZ* ^‐/‐^ flies respond normally to cardiac pacing. (A). There was no significant difference in percent failure between *TAZ* ^+/+^ flies (black, *n* = 102) and *TAZ* ^−/−^ flies (gray, *n* = 74) at 1 week old (*P* = 0.318). (B) At 5 weeks old, there was no significant difference in percent failure between *TAZ* ^+/+^ flies (black, *n* = 95) and *TAZ* ^−/−^ flies (gray, *n* = 82). (C) The difference between *TAZ* ^+/+^ (black, *n* = 95) and *TAZ* ^−/−^ (gray, *n* = 90) on the 5% sugar diet was not significant (*P* = 0.4648). (D) Exercise did not provide cardio protection in *TAZ* ^−*/*−*‐*^ EX flies (checkered pattern, *n* = 85) with the difference between *TAZ* ^−/−^ EX and *TAZ* ^−/−^ UN (gray, *n* = 80) being insignificant (*P* = 0.956). Significance was determined by an *F*‐Test for binary measures.

Cardiac failure rate after pacing was identical between 1‐week‐old *TAZ* ^*‐/‐*^ mutants and controls (Fig. 5A). We also detect no evidence of a progressive cardiac phenotype in *TAZ* mutants, as 5‐week‐old *TAZ* mutants also have identical failure rate to controls after pacing (Fig. 5B). Both mutants and controls increased failure rate with age, as is commonly observed (Wessells et al. 2004). This phenotype was also unaffected by reduced glucose availability (Fig. 5C). Typically, exercise improves ability to resist cardiac pacing stress (Sujkowski et al. 2015, 2017). However, after endurance exercise training, exercised *TAZ* mutants and unexercised *TAZ* mutants displayed similar failure rate, indicating no resistance to cardiac pacing was gained from endurance training (Fig. 5D).

## Discussion

Most patients diagnosed with Barth syndrome are widely recognized by pronounced skeletal myopathy, low muscle mass, delayed gross motor development, exercise intolerance, and muscle weakness (Barth et al. 1983; Spencer et al. 2006; Thompson et al. 2016). Skeletal myopathy leads to easy fatigability, which is exaggerated by the cardiovascular complications associated with Barth syndrome (Spencer et al. 2011). For the first time, we demonstrated that *Drosophila TAZ* mutants exhibit reduced endurance, climbing speed, and failure to adapt to a program of chronic exercise. As previously reported, physical impairment was observed in *TAZ* ^−/−^ flies’ climbing ability and flight ability (Xu et al. 2006a). Our results support the idea that *Drosophila TAZ* mutants experience weakened muscle function that is not improved with endurance exercise training.

This impairment is most likely due to impaired mitochondrial function, as *TAZ* mutants have been shown to have fragmented mitochondria with abnormal cristae morphology (Xu et al. 2006a). Mitochondrial function is critical for baseline endurance and for exercise adaptations in *Drosophila*. Chronic exercise increases mitochondrial turnover and decreases the percentage of oxidized mitochondria in muscle (Laker et al. 2014). In addition, it is possible to increase the endurance of a wild‐type fly line by introgressing mitochondria from a high‐performance fly line (Sujkowski and Wessells, unpublished observation). Thus, it is logical that mitochondrial dysfunction would reduce endurance and exercise adaptations in flies, as it does in vertebrate models (Powers et al. 2013) and humans (Bashir et al. 2017; Cade et al. 2017).

Barth Syndrome patients often have cardiac phenotypes that substantially affect quality of life (Spencer et al. 2006). Unexpectedly, adult *TAZ* ^−/−^ flies did not display any significant difference in cardiac stress resistance relative to *TAZ* ^+/+^ regardless of diet or age. *TAZ* mutant larvae also have no difference in resting heart rate (Xu et al. 2006a), suggesting that the heart complications resulting from loss of *tafazzin* function in vertebrates may not be present in insects. Interestingly, the normal cardiac function in this model gives us a unique opportunity to study exercise intolerance in *TAZ* mutants without the confounding factor of an impaired cardiac system. This study establishes an exercise intolerance phenotype for *Drosophila TAZ* mutants, and opens the door for future work to use endurance as a screening tool to identify physiologically relevant genetic interactors for *TAZ*.

## Conflict of Interest

None to declare.
